# COVID-19 positive test result from a private hospital laboratory: Neglecting to report and problems with national infection control

**DOI:** 10.1017/ice.2020.93

**Published:** 2020-03-27

**Authors:** Won Sriwijiatalai, Viroj Wiwanitkit

**Affiliations:** 1 TWS Medical Center, Bangkok, Thailand; 2 Dr DY Patil University, Pune, India; 3 Hainan Medical University, Haikou, China

*To the Editor*—The new coronavirus disease (COVID-19) is a new global public health problem. It already affects more than 140 countries around the world. Thailand is the second country in the timeline of disease pandemic.^1^ The infection has occurred since early January 2020, and COVID-19 remains uncontrollable problem. The Thai Ministry of Public Health tried several methods to counteract the disease outbreak. As a legal control, COVID-19 is included on the national list of infectious diseases under surveillance. The law requires that when a medical center detects this disease, an official reports it to the governmental center for disease control (CDC) within 3 hours. Violation of this legal control should result in a punitive consequence.

The exact advantage of this legal regulation regarding COVID-19 control is an interesting issue. In Thailand, not only governmental hospitals but also some private hospitals have the ability to conduct laboratory diagnosis of COVID-19. Among 82 cases of COVID-19 in Thailand overall (as of March 15, 2020), in 1 interesting case the patient self-declared infection via Instagram but no official report had been filed with the local CDC. This patient had received a positive laboratory result from a private hospital. This case was finally confirmed as a case of COVID-19, but it appears that the private hospital may have neglected to report it, violating the legal requirement for disease reporting. This example represents a big challenge faced by hospitals in infection control. Good data collection is important to obtaining high-quality surveillance data, which is imperative for infectious disease control.^2^ At times, poor, unethical, private hospitals do not follow the disease control guidelines. In a disease outbreak, collaboration from hospitals is required, and strict legal regulation of the disease control system should be undertaken. In cases of violation, the poor role model should be punished.
